# Utilizing the Fungal Bicistronic System for Multi-Gene Expression to Generate Insect-Resistant and Herbicide-Tolerant Maize

**DOI:** 10.3390/ijms252413408

**Published:** 2024-12-14

**Authors:** Yuxiao Chen, Wenjie Lv, Qun Yue, Ning Wen, Yinxiao Wang, Zhihong Lang, Wei Xu, Shengyan Li

**Affiliations:** 1 College of Plant Protection, Jilin Agricultural University, Changchun 130118, China; chenyuxiao0814@163.com (Y.C.); 15656887881@163.com (W.L.); 2 Biotechnology Research Institute, Chinese Academy of Agricultural Sciences, Beijing 100081, China; yuequn@caas.cn (Q.Y.); 82101211020@caas.cn (N.W.); wangyinxiao@caas.cn (Y.W.); langzhihong@caas.cn (Z.L.); 3 National Nanfan Research Institute (Sanya), Chinese Academy of Agricultural Sciences, Sanya 572025, China

**Keywords:** *IGG6*, bicistron, insect resistance, glyphosate tolerance, maize

## Abstract

Developing simple and efficient multi-gene expression systems is crucial for multi-trait improvement or bioproduction in transgenic plants. In previous research, an *IGG6*-based bicistronic system from the nonpathogenic fungus *Glarea lozoyensis* efficiently expressed multiple enzyme proteins in yeast and maize, and the heterologous enzymes successfully performed their catalytic activity to reconstruct the biosynthetic pathway in the host organism. Unlike enzyme proteins, some heterologous functional proteins (such as insecticidal proteins) are dose-dependent and they need to express sufficient levels to perform their biological functions. It remains unclear whether the *IGG6*-based bicistronic system can achieve high expression of the functional proteins for practical applications in crops. In this study, two *Bacillus thuringiensis* (Bt) insecticidal genes, *vip3Aa* and *cry1Ab*, were linked via *IGG6* to form a bicistron, while two glyphosate resistance genes, *gr79epsps* and *gat*, served as monocistronic selectable marker genes. Regenerated maize plants were produced through genetic transformation. RNA and immunoblot analyses revealed that the *vip3Aa*-*IGG6*-*cry1Ab* bicistron was transcribed as a single transcript, which was then translated into two separate proteins. Notably, the transcription and translation of *cry1Ab* were significantly positively correlated with those of *vip3Aa*. Through ELISA and leaf bioassay, we identified two transgenic maize lines, VICGG-15 and VICGG-20, that exhibited high insecticidal activity against fall armyworm (FAW; *Spodoptera frugiperda*) and Asian corn borer (ACB; *Ostrinia furnacalis*), both of which had high expression of Vip3Aa and Cry1Ab proteins. Subsequent evaluations, including silk, ear, and field bioassays, as well as glyphosate tolerance assessments, indicated that the VICGG-15 plants displayed high resistance to FAW and ACB, and could tolerate up to 3600 g acid equivalent (a.e.) glyphosate per hectare without adversely affecting phenotype or yield. Our finding established that the *IGG6*-based bicistronic system can achieve high expression of functional proteins in maize, and it is a potential candidate for multi-gene assembly and expression in plants.

## 1. Introduction

After nearly 30 years of development, genetically modified (GM) crops have significantly contributed to increasing food production, raising farmers’ incomes, and protecting the environment, establishing themselves as the most rapidly adopted biotechnology [1]. However, as food demand continues to grow alongside the frequent occurrence of major pests, diseases, and extreme weather events, single-gene and single-trait GM crops are no longer able to meet the needs of modern agriculture. Therefore, multi-gene and multi-trait GM crops are becoming the future direction of GM crops development. The simultaneous expression of multiple genes in crops, which requires a promoter and terminator for each coding sequence (CDS), significantly complicates the design of genetic constructs, their generation via cloning, and their introduction into target species through genetic transformation. Currently, the GM crop is usually obtained by using conventional hybridization methods to combine advantageous genes/traits. For example, Genuity SmartStax was obtained by crossing four transgenic maize events (MON89034, TC1507, MON88017, and DAS-59122), resulting in the expression of the following eight foreign genes: six insecticidal genes (*cry2Ab2*, *cry1A.105*, *cry3Bb1*, *cry1Fa2*, *cry34Ab1*, and *cry35Ab1*) and two herbicide resistance genes (*cp4epsps* and *pat*). However, this approach is complex, time-consuming, and inefficient. Thus, developing and improving methods for multi-gene stacking in plants is an expanding and exciting field of current research [2]. In the previous study, different multi-gene expression systems have been proposed: (1) pre-mRNA splicing; (2) proteolytic cleavage sites; (3) fusion proteins; (4) internal ribosomal entry sites (IRESs); and (5) self-cleaving 2 A peptides [3,4,5,6,7]. Currently, the most widely used systems for the expression of multiple genes from a single transgene are IRESs and 2A peptides [4,6,8,9]. However, the IRESs have a large size (typically over 500 nucleotides) and lower expression levels of the downstream gene [7]. The viral origin of 2A peptides may raise regulatory concerns and the addition of amino acids to the terminus of the proteins may have adverse effects on their structures and functions [10,11]. These disadvantages limit their application.

In our previous research, a short intergenic sequence (*IGG1*) was identified that connects separate genes expressed as a functional polycistronic operon in the filamentous fungus *Glarea lozoyensis* [12]. A 9-nt nucleotide sequence, named *IGG6*, was obtained by optimizing *IGG1*. Using the *IGG6* bicistronic system, we achieved the expression of multiple exogenous enzyme proteins and detected corresponding products in both yeast and maize [11,13]. Although we demonstrated that the *IGG6*-based bicistronic system could be used for expressing multiple enzyme proteins in yeast and maize, its suitability for the expression of dose-dependent functional proteins, such as Bt insecticidal protein, remained unclear.

In this study, we tested the suitability of the *IGG6* bicistronic system by using two Bt insecticidal genes, *vip3Aa* and *cry1Ab*, which had high insecticidal activity against FAW and ACB, respectively. Meanwhile, two glyphosate resistance genes (*gr79epsps* and *gat*) in the form of monocistrons were used as control and selectable marker genes. Through qRT-PCR, ELISA, and bioassays, we further demonstrated the mechanism of the *IGG6*-based bicistronic system and determined the efficiency of the *IGG6* bicistronic system for expressing dose-dependent functional proteins. This research contributes to a deeper understanding of the *IGG6*-based bicistronic system and highlights its potential as a promising tool for multi-gene assembly and expression, which could be introduced into crops through genetic modification.

## 2. Results

### 2.1. Maize Transformation and Identification of Transgenic Maize Plants

**Figure 1:**
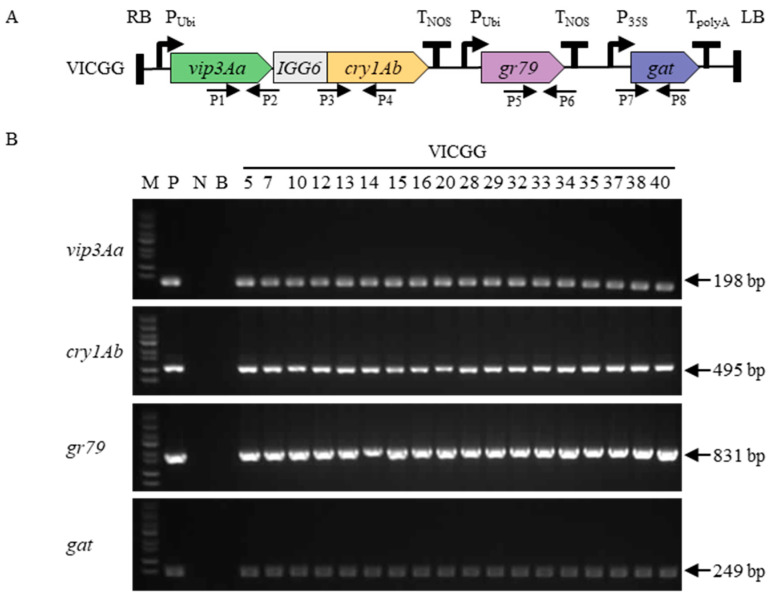
PCR screening of regenerated maize plants. (**A**) Schematic diagram of the maize genetic transformation constructs VICGG. The glyphosate resistance genes *gat* and *gr79-epsps* were used as selected marker genes for maize transformation. The black arrow represents the primer position. (**B**) PCR analysis of the *vip3Aa*, *cry1Ab*, *gr79,* and *gat* genes in corresponding transgenic maize plants.

Agrobacterium tumefaciens strain EHA105 carrying VICGG vector was used for the transformation of maize inbred line B104 immature zygotic embryos (Figure 1A). A total of 40 regenerated maize plants were obtained, and PCR screening confirmed that 31 of them carried the four genes (*vip3Aa*, *cry1Ab*, *gr79epsps*, and *gat*). The results of PCR analysis for the four genes in the corresponding transgenic maize plants are shown in Figure 1B. This result confirmed that the exogenous genes were successfully transformed into the maize plants.

### 2.2. Quantitative Analysis of Protein Content and Transcription Level in Transgenic Maize Plants

**Figure 2:**
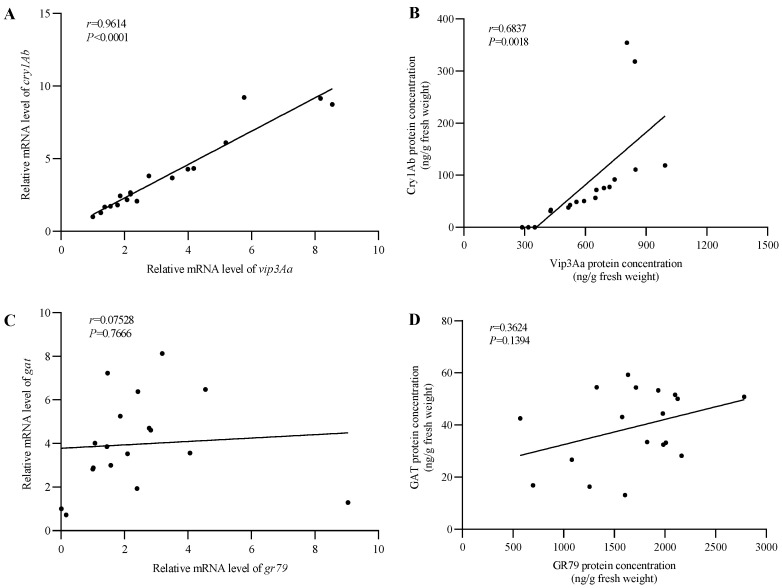
Correlation between *cry1Ab* and *vip3Aa*, as well as *gat* and *gr79*, at the transcription and translation levels in the 18 VICGG transgenic maize lines. (**A**,**B**) Correlation between *cry1Ab* and *vip3Aa* at the transcription and translation levels. (**C**,**D**) Correlation between *gat* and *gr79* at the transcription and translation levels. All data were presented as the mean of three biological replicates. Pearson’s correlation coefficients and their statistical significance were determined using GraphPad prism.

The accumulation of each target protein (Vip3Aa, Cry1Ab, GR79EPSPS, and GAT) in the transgenic maize lines was quantified by ELISA. Quantitative analysis of Vip3Aa protein content revealed that 18 of the VICGG transgenic maize lines expressed the Vip3Aa protein and the content of Vip3Aa protein varied from 287.83 to 993.46 ng g^−1^ fresh weight (Table S2). Subsequently, the protein expression levels of Cry1Ab, GR79EPSPS, and GAT in these 18 transgenic maize lines were assessed. The content of Cry1Ab, GR79EPSPS, and GAT protein varied from 31.59 to 354.28 ng g^−1^ fresh weight, 571.05 to 2781.90 ng g^−1^ fresh weight, and 13.19 to 59.32 ng g^−1^ fresh weight, respectively (Table S2). The relative mRNA levels of the four target genes in the 18 VICGG lines were detected by qRT-PCR. The qRT-PCR results showed that all four target genes were successfully transcribed in the 18 VICGG lines and the transcription levels were varied among the different transgenic maize lines (Table S3). The statistical analysis indicated that the expression of *cry1Ab* gene was significantly positively correlated with *vip3Aa* (transcription level correlation analysis: r = 0.9614 *p* < 0.0001, translation level correlation analysis: r = 0.6837, *p* = 0.0018) (Figure 2A,B), while there was no correlation between the *gr79epsps* and *gat* genes (transcription level correlation analysis: r = 0.07528 *p* = 0.7666, translation level correlation analysis: r = 0.3624, *p* = 0.1394) (Figure 2C,D).

### 2.3. The Transcript and Protein Forms of the IGG6-Based Bicistron

**Figure 3:**
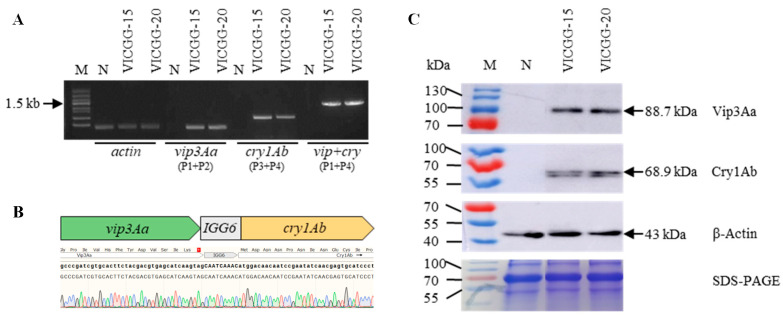
RT-PCR and immunoblot analysis of the transcript and protein forms of *IGG6*-mediated bicistron in VICGG plants. (**A**) RT-PCR analysis of the transcript forms of *IGG6*-mediated bicistron. Primers P1/P2, P3/P4 and P1/P4 were used to detect the *vip3Aa*, *cry1Ab* and the bicistronic mRNA, respectively. The maize *actin1* gene was used as control. M: Trans5K DNA Marker, P: positive control, N: wild-type maize plants, B: blank. (**B**) The BLAST result of the bicistronic mRNA’s PCR amplicon. (**C**) Immunoblot analysis of the sizes of Vip3Aa and Cry1Ab protein. The β-Actin protein was used as control. M: PageRuler^TM^ prestained protein ladder,10 to 180 kDa. N: wild-type maize plants.

To confirm the transcript and protein forms of the *vip3Aa-IGG6-cry1Ab* bicistron, we chose two transgenic maize lines (VICGG-15 and VICGG-20) and performed RT-PCR and immunoblot analysis of the transgenic maize plants. For RT-PCR analysis, we used two pairs of primers (P1/P2 and P3/P4) to amplify *vip3Aa* and *cry1Ab* individually. We also detected the bicistronic mRNA with the forward primer P1 for *vip3Aa* (the first CDS) and the reverse primer P4 for *cry1Ab* (the second CDS). The results of RT-PCR showed that the *vip3Aa* and *cry1Ab* mRNA could be detected, as well as the *vip3Aa-IGG6-cry1Ab* bicistronic transcript, in transgenic maize plants (Figure 3A). All RT-PCR amplicons were subjected to Sanger sequencing to confirm their identities (Figure 3B). For immunoblot analysis, we used anti-Vip3Aa and anti-Cry1Ab antibodies to assess the sizes of the immunoreactive proteins in transgenic maize plants. The expected molecular weight of Vip3Aa is 88.7 kDa, while that of Cry1Ab is 68.9 kDa; a fusion protein between Vip3Aa and Cry1Ab would have a predicted molecular weight of 160.4 kDa. We detected a single band when using the anti-Vip3Aa or anti-Cry1Ab antibodies against protein extracts from VICGG-15 and VICGG-20 plants, with no Vip3Aa or Cry1Ab detected in B104 plants. Importantly, the size of Vip3Aa or Cry1Ab detected in VICGG transgenic maize plants was consistent with the predicted size of each protein (Figure 3C). These observations strongly suggested that the *IGG6*-based bicistron was transcribed as a single transcript rather than two separate transcripts and translated as separate proteins rather than as a single fusion protein.

### 2.4. Bioassay of VICGG Transgenic Maize Lines

**Figure 4:**
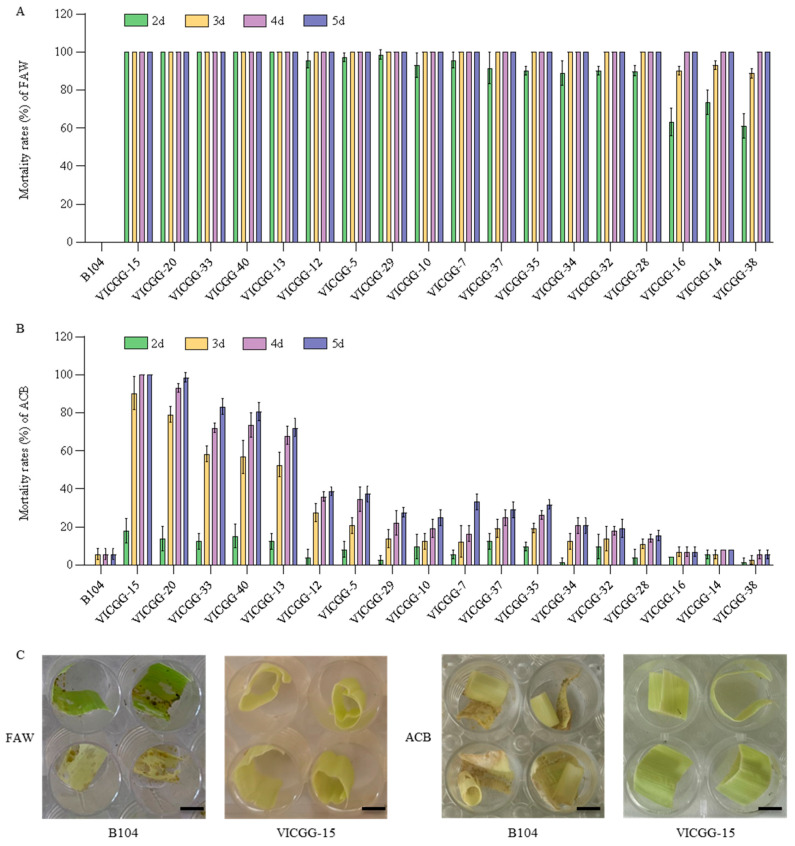
Laboratory bioassays of VICGG transgenic maize leaves with fall armyworm (FAW) and Asian corn borer (ACB). (**A**,**B**) The mortality rates of FAW and ACB larvae feeding on the leaves of wild-type and VICGG transgenic maize plants. Data represent means ± SD (n = 3 biological replicates). (**C**) The appearance of wild-type and transgenic maize leaves after insect bioassays with FAW and ACB. Photographs were taken after 5 days of infestation. Scale bar = 0.5 cm.

**Figure 5:**
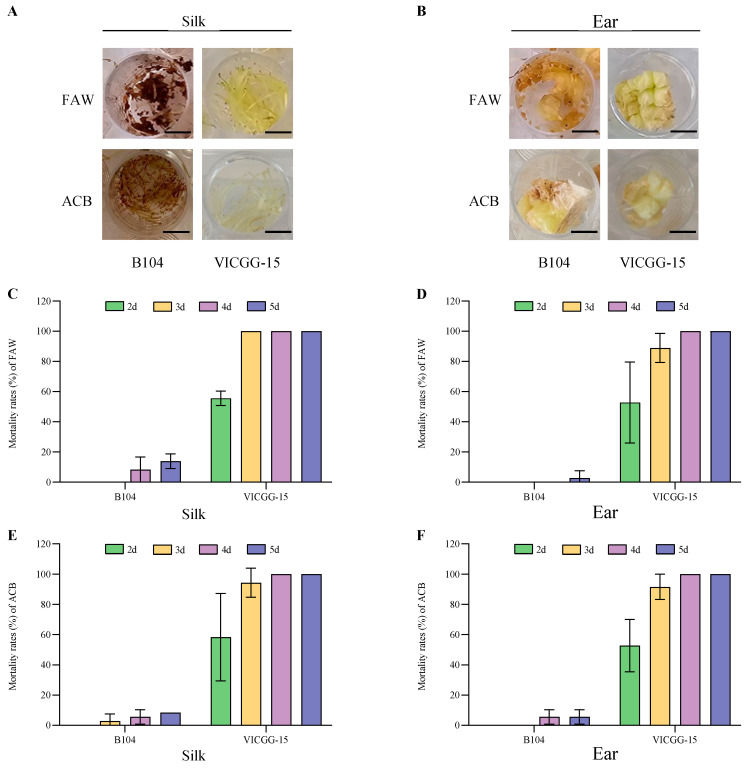
Laboratory bioassays of VICGG-15 transgenic maize silk and ear with fall armyworm (FAW) and Asian corn borer (ACB). (**A**,**B**) The appearance of wild-type and VICGG-15 transgenic maize silks and ears after insect bioassays with FAW and ACB. Photographs were taken after 5 days of infestation. Scale bar = 0.5 cm. (**C**,**D**) The mortality rates of FAW larvae feeding on the silks and ears of wild-type and VICGG-15 plants. (**E**,**F**) The mortality rates of ACB larvae feeding on the silks and ears of wild-type and VICGG-15 plants. Data represent means ± SD (n = 3 biological replicates).

To determine whether the Vip3Aa and Cry1Ab proteins produced from the *IGG6*-based bicistron have insecticidal activity against their target insects, we evaluated the resistance of 18 VICGG transgenic maize lines to FAW and ACB in laboratory bioassays, using nontransgenic B104 plants as control. We recorded the number of living and dead larvae every day, from which we calculated the mortality rates of FAW and ACB larvae; we photographed the extent of leaf damage on day 5. After 5 days of infestation with the neonate larvae, the leaves of all VICGG lines showed significant resistance to FAW compared to wild-type B104 plants (Figure 4A), while only VICGG-15 and VICGG-20 lines showed significant resistance to ACB with the mortality rates of ACB larvae of 100% and 98.61% (Figure 4B). The leaves of VICGG-15 exhibited slight damage, and all FAW and ACB larvae died. In contrast, the leaves of B104 were badly damaged and most of the larvae survived (Figure 4C). Consistent with the results of the leaf bioassays, the silks and ears of VICGG-15 plants showed significant resistance to FAW and ACB compared to those of B104 plants (Figure 5).

**Figure 6:**
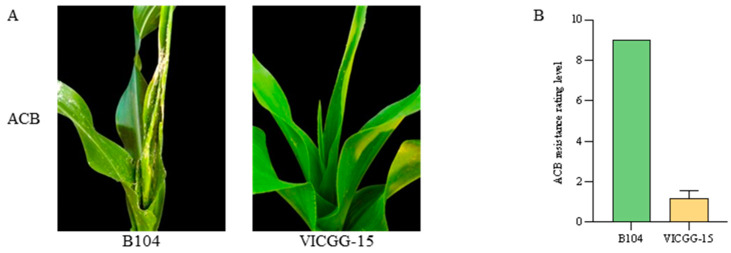
Field bioassays of VICGG-15 transgenic maize plants with Asian corn borer (ACB). (**A**) The appearance of wild-type and VICGG-15 transgenic maize plants after insect bioassays with ACB. Photographs were taken after 3 days of infestation. (**B**) The resistance rating level of wild-type and VICGG-15 transgenic maize plants to ACB. Data represent means ± SD (n = 30).

Based on the results of the laboratory bioassays described above, we chose the VICGG-15 line to assess the resistance of transgenic maize plants to ACB in field bioassays. After 3 days of infestation with the 3rd instar larvae, the VICGG-15 plants showed high resistance to ACB and the leaves exhibited slight damage, while the leaves of B104 plants were badly damaged (Figure 6A). The average resistance rating level of VICGG-15 plants to ACB was 1.17, reaching a high resistance (HR) level. On the contrary, the average resistance rating level of B104 plants to ACB was 9.0, reaching a high susceptible (HS) level (Figure 6B). We conclude that the VICGG-15 plants were toxic to both FAW and ACB.

### 2.5. Glyphosate Tolerance Analysis and Agronomic Traits Investigation of Transgenic Maize VICGG-15

**Figure 7:**
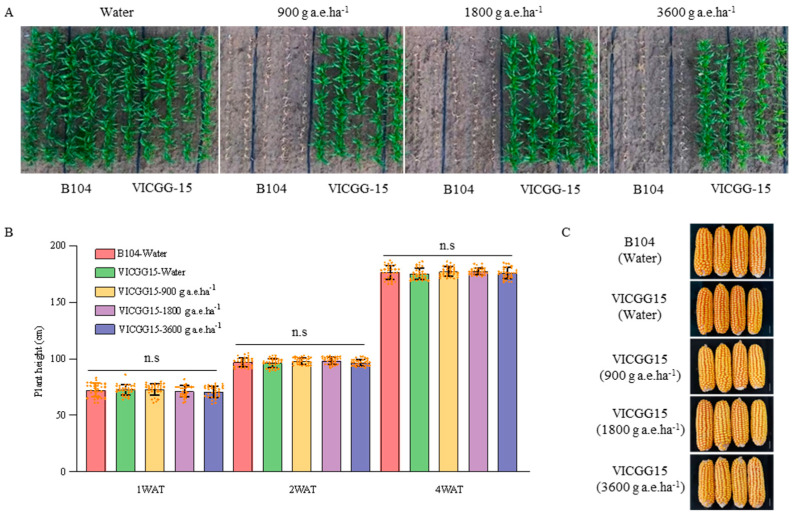
Glyphosate tolerance analysis and agronomic traits investigation of VICGG-15. (**A**) Pictures of VICGG-15 and B104 (WT) plants were recorded 10 days after treatment with glyphosate (900, 1800, and 3600 g a.e.ha^−1^), water as negative control. Aerial view taken by Unmanned Aerial Vehicle. (**B**) The plant height of VICGG-15 at 1, 2, and 4 weeks after glyphosate treatment. Data are means ± SD (n = 30), n.s: no significance (*p* > 0.05, one-way ANOVA). (**C**) Ear phenotype of B104 and VICGG-15. Scale bar = 2 cm.

The VICGG-15 plants not only highly expressed the insecticidal proteins of Vip3Aa and Cry1Ab but also produced the GR79EPSPS and glyphosate N-acetyltransferase. Compared to the previously obtained glyphosate-resistant transgenic maize event GG2 [14], the contents of GR79EPSPS and GAT proteins in both were comparable. Therefore, we also evaluated its glyphosate resistance level in the field. The results of glyphosate tolerance analysis showed that all B104 plants were dead from being treated with any dose of glyphosate after one week, whereas there was no injury symptom on VICGG-15 plants (Figure 7A). The plant height and seedling survival rate of glyphosate-treated VICGG-15 plants were not significantly different from those treated with water only at 1, 2, or 4 WAT (Figure 7B and Table S4). The results of the investigation showed that the main agronomic traits of VICGG-15 plants treated with glyphosate were also not significantly different from those of B104 at the R6 stage (Figure 7C and Table S5). This observation indicates that the transgenic maize VICGG-15 can tolerate high doses of glyphosate with no negative effect on agronomic traits.

## 3. Discussion

### 3.1. Mechanism and Efficiency of IGG6-Based Bicistronic System

Polycistronic RNAs are common in prokaryotes but rarely exist in eukaryotes. With the development of long-read sequencing and the exponential accumulation of genomic data, more polycistronic genes are being discovered in eukaryotic species [15,16,17]. The eukaryotic polycistronic loci reported to date can be divided into two types: the first type is transcribed into polycistronic transcripts that are subsequently processed into mature monocistronic mRNAs; the second type is transcribed into mature polycistronic mRNA that is translated as individual proteins. In this study, we found the *IGG6*-based bicistron was transcribed in maize as a single transcript and then translated into two separate proteins. Therefore, the *IGG6*-based polycistron belongs to the second type. Two mechanisms drive the translation of several proteins from a single polycistronic transcript: post-termination reinitiation [18] and leaky ribosomal scanning [19]. In our previous study, we found that increasing the number of stop codons (from one-stop codons to three-stop codons) following the first CDS in the *IGG6*-based bicistronic system significantly reduced the protein expression of the second CDS. When the first CDS ended with three stop codons, the second protein was undetectable [13]. Moreover, the 9-nt region of *IGG6* is insufficient in length to recruit ribosomes in the same manner as an IRES sequence [20]. Therefore, we infer that the translation mechanism of *IGG6*-based bicistronic system is more consistent with the “post-termination reinitiation” mechanism. In other words, instead of disassembling after translating the first protein, the ribosomal complexes move to the start codon of the second gene and resume translation in the *IGG6*-based bicistronic system.

An advantage of polycistronic mRNAs is the possibility of translational coupling of two or more genes, whereby the translation of a downstream gene depends on the translation of the upstream gene [21]. In this study, we found the transcription and translation of the downstream *cry1Ab* gene were coupled to the upstream *vip3Aa* gene in the *IGG6*-based bicistronic system. The expression of downstream Cry1Ab protein was 9.5% to 56.6% of that of upstream Vip3Aa in transgenic maize plants. In our previous study, the accumulation of GFP was from 31% to 76% of the different upstream drive proteins in *S. cerevisiae* [11] and the fluorescence intensity of GFP from mCherry-*IGG6*-GFP was 65.81% of that from GFP-*IGG6*-mCherry in maize protoplasts [13]. Compared with the results from yeast and maize protoplasts, the efficiency of *IGG6* was lower in this study, which may be related to the longer length of the *vip3Aa* gene. Some studies have shown that post-termination reinitiation efficiency decreases with increasing length of the upstream ORF (uORF) [8,22].

### 3.2. Application Prospect of IGG6 in GM Crops

In this study, and our previous research, we have demonstrated that the *IGG6*-based bicistronic system could be used for the expression of different types of genes. Compared to IRES and 2A, the expression efficiency of downstream genes in the *IGG6*-based bicistronic system was slightly lower than that of 2A, but much higher than that of IRES [11]. In terms of size, the *IGG6* (only 9 nucleotides) was much smaller than both IRES (typically over 500 nucleotides) [7] and 2A (18–22 amino acids) [23]. This made *IGG6* more convenient for genetic manipulation and allowed for the easier assembly of more genes in the same space. In protein translation, the *IGG6*-based bicistronic system did not add any extra amino acids that could affect the function of the protein. Finally, in terms of biosafety, compared to the viral origin of IRES and 2A peptides, the *IGG6*-based bicistronic system offered higher safety and does not raise regulatory or public concerns, as it was sourced from the nonpathogenic fungus *Glarea lozoyensis*. Therefore, *IGG6*-mediated multi-gene expression had more distinct advantages in GM crops.

In GM crops, the realization of a specific metabolic pathway often requires the coordinated expression of multiple genes (typically more than 10). Although the *IGG6* system can reduce the number of genetic elements, it still faces the problem of a lack of genetic elements when expressing a larger number of genes. In the *IGG6*-based bicistronic system, the transcription and translation of downstream genes are coupled with the upstream gene. Based on this, we developed a strategy termed HACKing (highly efficient and accessible system by CracKing genes into the genome) for assembling multi-gene metabolic pathways by combining CRISPR/Cas9-based genome editing. We successfully expressed 13 exogenous genes in yeast, leading to the production of mogrol [11]. Although it is not yet possible to achieve such large-scale gene knock-in in GM crops, it is believed that with the rapid development of gene editing technologies, the *IGG6* system will play a pivotal role in the development of multi-gene and multi-trait GM crops.

## 4. Materials and Methods

### 4.1. Construction of Vectors

The intergenic sequence *IGG6* (5′-CAATCAAAC-3′) [11] was synthesized by GenScript Biotech Corporation (Nanjing, China). The maize *Ubiquitin* promoter, insecticidal genes (*vip3Aa* and *cry1Ab*), and *NOS* terminator were amplified from our laboratory plasmids. These DNA fragments were mixed in the appropriate combinations and ligated into *Hind* III-digested pCGG [14] by seamless assembly cloning (Thermo Fisher Scientific, A14603) to generate the maize transformation vector: VICGG (*Ubipro:vip3Aa-IGG6-cry1Ab:NOS*; *Ubipro:gr79epsps:NOS; 35S:gat:polyA*). The glyphosate resistance genes *gat* (*glyphosate N-acetyltransferase*) and *gr79epsps* (*5-enolpyruvylshikimate-3-phosphate synthase*) were sourced from our laboratory and used as selectable marker genes for maize transformation.

### 4.2. Maize Transformation

The VICGG vectors were transformed into immature zygotic maize embryos by *Agrobacterium* (*Agrobacterium tumefaciens* strain EHA105)-mediated transformation. *Agrobacterium*-mediated transformation and regeneration of transgenic plants were performed as previously described [24]. Positive calli and regenerated plantlets were selected on Murashige and Skoog medium containing glyphosate (0.2 g L^−1^). All plant tissue culture reagents were purchased from PhytoTechnology Laboratories (Shawnee Mission, KS, USA). The immature zygotic maize embryos of B104 and *Agrobacterium tumefaciens* strain EHA105 used for maize transformation were both sourced from our laboratory.

### 4.3. Molecular Analysis of Transgenic Maize Plants

Genomic DNA was extracted from maize leaves using the CTAB method [25]. Transgenic maize plants were confirmed by PCR with gene-specific primers. For RT-PCR and qRT-PCR analysis of transgenic maize plants, total RNA was extracted from fresh maize leaves using TRIzol reagent (R401, Vazyme, Nanjing, China). Approximately 1 μg of total RNA was used as a template for reverse transcription into first-strand cDNA using a RevertAid First Strand cDNA Synthesis Kit (K1622, Thermo Fisher Scientific, Waltham, MA, USA). Relative gene expression levels were calculated using the delta CT method; maize Actin1 (Zm00001d010159) was used as the reference gene. All primers are listed in Table S1.

Enzyme-linked immunosorbent assay (ELISA) kits for Vip3Aa (AA1641, YouLong Biotech, Shanghai, China), Cry1Ab (AA0341, YouLong Biotech, Shanghai, China), GR79EPSPS (AA1941, YouLong Biotech, Shanghai, China) and GAT (AA1341, YouLong Biotech, Shanghai, China) were used to detect the amounts of Vip3Aa, Cry1Ab, GR79EPSPS and GAT in maize plants following the manufacturer’s protocols. The optical density was measured at 450 nm using a BioTek Elx808 microplate reader (BioTek Instruments, Winooski, VT, USA). For immunoblot analysis, total soluble proteins were extracted from maize leaves in phosphate-buffered saline containing 0.05% (*v*/*v*) Tween-20 (PBST). The following experimental procedures were performed as previously described [26]. The anti-Vip3Aa (AA1624, YouLong Biotech, Shanghai, China), anti-Cry1Ab (AbM59701-5-PU, Beijing Protein Innovation, Beijing, China), and anti-Actin (BP0101, Lablead, Beijing, China) primary antibodies were used at a 1:5,000 dilution; an HRP-conjugated goat anti-mouse (CW0102, CWBIO, Beijing, China) secondary antibody was used at a 1:10,000 dilution. The signals were detected in an Amersham Imager 600 (GE Healthcare, Chicago, IL, USA) using an eECL Western blot kit (CW0049, CWBIO, Beijing, China).

### 4.4. Laboratory Bioassays

The resistance of transgenic maize plants to FAW and ACB was evaluated using leaves, silks, and immature ears. Wild-type maize plants (inbred line B104) were used as a control. Culture plates with 12 or 24 wells (3513 or 3524, Corning, Kennebunk, ME, USA) were used as rearing containers for the insects. At the V6 stage, the youngest leaves were cut into 1 cm segments and placed into each well of a 24-well plate. At the R1 stage, silks and immature ears were cut into segments or small pieces and placed into each well of a 12-well plate. One neonate larva was placed into each well. For leaves, 24 larvae in a 24-well plate were used as one treatment. For silks and ears, 12 larvae in a 12-well plate were used as one treatment. We performed three biological replicates with each treatment. All plates were incubated in a rearing room at 70–80% relative humidity, 26 °C, and a photoperiod of 16 h light/8 h dark. The number of living and dead larvae was recorded every day for 7 days. Mortality rates are presented as the proportion of dead larvae to total larvae (%). The inbred line B104 and transgenic maize plants were sourced from our laboratory. The neonate larvae of FAW and ACB were purchased from MeiYan (Beijing) Agricultural Technology Co., Ltd. (Beijing, China).

### 4.5. Field Bioassays

Due to the fact that fall armyworm is an invasive species, the resistance of transgenic maize plants to ACB was only evaluated in the field bioassays, with B104 plants as control. At the V8 stage, each maize plant was infested with 20 third instar larvae of ACB at 6:00 p.m. We performed thirty biological replicates with each treatment. Leaf damage was estimated through visual inspection after 3 days of infestation. The leaf damage was classified using a severity scale of 1–9 according to the Rules for Evaluation of Maize for Resistance to Asian corn borer [27]. The 3rd instar larvae of ACB were purchased from MeiYan (Beijing) Agricultural Technology Co., Ltd.

### 4.6. Glyphosate Tolerance Analysis

Commercially formulated isopropylamine salt of glyphosate (Roundup; Bayer, Leverkusen, Germany) was used to assess the glyphosate resistance levels of transgenic maize plants according to the evaluation of the environmental impact of genetically modified plants and its derived products of herbicide-tolerant maize (Part 1: Evaluation of the Tolerance to Herbicides). B104 and transgenic maize plants were sprayed with different doses of glyphosate (900, 1800, or 3600 g a.e. ha^−1^) at the six-leaf stage, using water as the negative control. The survival rate, plant height, and injury symptoms of all plants were recorded at 1, 2, and 4 weeks after treatment (WAT). The agronomic traits were investigated and recorded at the R6 stage.

### 4.7. Agronomic Traits Investigation

The agronomic traits of maize, including plant height, ear height, ear length, ear diameter, row numbers per ear, kernels per row, bald tip length, hundred-kernel weight, and yield per plant were investigated and analyzed.

### 4.8. Statistical Analysis

All data were analyzed using Excel 2021 (Microsoft Corporation, Redmond, WA, USA), SAS 9.4 (SAS, Cary, NC, USA) and GraphPad Prism 9.4 (GraphPad Software, Boston, MA, USA).

## 5. Conclusions

In the present study, we selected two insecticidal genes to evaluate whether the *IGG6*-based bicistronic system was suitable for the expression of functional proteins in maize, along with two monocistronic glyphosate resistance genes as selection marker genes. Transcript and protein detection results showed that the *IGG6*-based bicistron was transcribed as a single transcript and then translated into separate proteins. Furthermore, the transcription and translation of the downstream genes were coupled with the upstream gene, which was consistent with our previous findings. Through bioassay analysis, we obtained a transgenic event, VICGG-15, from 18 VICGG transgenic maize lines, showing high insecticidal activity to FAW and ACB. Glyphosate tolerance analysis and agronomic trait investigation also demonstrated that VICGG-15 exhibited high glyphosate resistance without any negative impact on agronomic traits. In conclusion, our findings confirm that the *IGG6*-based bicistronic system is effective for the expression of functional proteins, and using this system, we successfully obtained a transgenic maize event with high insect resistance and glyphosate tolerance, providing a foundation for the development of multi-gene and multi-trait GM crops.

## Data Availability

The original contributions presented in this study are included in the article/Supplementary Material. Further inquiries can be directed to the corresponding authors.

## Supplementary Materials

The following supporting information can be downloaded at: https://www.mdpi.com/article/10.3390/ijms252413408/s1.

## Abbreviations

Abbreviation	full name
Bt	*Bacillus thuringiensis*
ELISA	enzyme-linked immunosorbent assay
FAW	fall armyworm
ACB	Asian corn borer
GM	genetically modified
IRESs	internal ribosomal entry sites
CDS	coding sequence
HR	high resistant
HS	high susceptible
WAT	weeks after treatment
