# Supplementary material for: Utilizing the Fungal Bicistronic System for Multi-Gene Expression to Generate Insect-Resistant and Herbicide-Tolerant Maize
Source: Int J Mol Sci. 2024 Dec 14;25(24):13408. doi: 10.3390/ijms252413408 (PMC11677970; doi:10.3390/ijms252413408)
Supplement: Supplementary file 1 [file ijms-25-13408-s001.zip › ijms-3325436-supplementary.pdf]

Table S1 Primers used in this study.

Primer name	Sequence	Purpose
35S-F	5'-TGCATGCCTGCAGGTCCCCA-3'	Primers for amplification of 35S promoter
35S-R	5'-GTCCCCCGTGTTCTCTCCAA-3'	
NLS-GFP-F	5'-ATGGATAAAGCGGAATTAAT-3'	Primers for amplification of *GFP* gene
NLS-GFP-R	5'-TTATTTGTATAGTTCATCCA-3'	
CTP-YFP-F	5'-ATGGCGCCCACCGTGATGAT-3'	Primers for amplification of *YFP* gene
CTP-YFP-R	5'-TTACTTGTACAGCTCGTCCA-3'	
NOS-F1	5'-GAATTTCCCCGATCGTTCAA-3'	Primers for amplification of NOS terminator
NOS-R1	5'-AATTCCCGATCTAGTAACAT-3'	
Ubi-F	5'-GCATGCCTGCAGTGCAGCGT-3'	Primers for amplification of *ubiquitin* promoter
Ubi-R	5'-GGATCCTCTAGAGTCGACCT-3'	
Vip3Aa-F1	5'-ATGAACAAGAACAACACCAA-3'	Primers for amplification of *vip3Aa* gene
Vip3Aa-R1	5'-CTACTTGATGCTCACGTCGT-3'	
Cry1Ab-F1	5'-ATGGACAACAATCCGAATAT-3'	Primers for amplification of *cry1Ab* gene
Cry1Ab-R1	5'-TCAGTACTCCGCCTCGAAAG-3'	
NOS-F2	5'-GGTACCGGGCCCCCCCTCGA-3'	Primers for amplification of NOS terminator
NOS-R2	5'-CCGATCTAGTAACATAGATG-3'	
P1	5'-GCATCAGCCAGTTCATCGG-3'	PCR, RT-PCR and qRT-PCR primers for detection of *vip3Aa* gene
P2	5'-ACACGCCCTTCAGGTCGGT-3'	
P3	5'-CGCATTGAGACGGGCTAC-3'	PCR and RT-PCR primers for detection of *cry1Ab gene*
P4	5'-CCTGGAGTTGATGGTGGC-3'	
P5	5'-TCGACGTGAACCCGATCAAC-3'	PCR, RT-PCR and qRT-PCR primers for detection of *gat gene*
P6	5'-TCTGCTCCCTGTAGCCCTCC-3'	
P7	5'-TCAGCAGGGCGAGTGGA-3'	PCR and RT-PCR primers for detection of *gr79 gene*
P8	5'-TCGTCGTGCGGGTTCAG-3'	
P9	5'-CGGCTTGCTACTTCCTCT-3'	qPCR primers for detection of *gat* gene
P10	5'-CGTTCGTCACCCTGATTG-3'	
P11	5'-CGCATTGAGACGGGCTAC-3'	qPCR primers for detection of *cry1Ab* gene
P12	5'-CGATCTGGACCAGGAACG-3'	
Actin-F1	5'-CGAATGCCCAGCAATGTA-3'	RT-PCR and qRT-PCR primers for detection of *actin1* gene
Actin-R1	5'-TTAGGTGGTCGGTGAGGT-3'	

Table S2 The Vip3Aa, Cry1Ab, GR79EPSPS and GAT proteins expression in VICGG transgenic maize lines.

Sample	Protein expression (ng/g fresh weight)			
	Vip3Aa	Cry1Ab	GR79EPSPS	GAT
VICGG-15	805.29±55.77	354.28±21.11	1325.66±145.11	54.50±2.48
VICGG-20	844.51±39.70	318.30±17.60	1636.34±139.43	59.32±2.27
VICGG-33	848.09±55.67	110.67±3.10	571.05±159.05	42.55±2.97
VICGG-40	744.60±48.06	91.92±4.79	2100.34±131.04	51.62±2.60
VICGG-13	993.46±79.49	118.94±2.42	697.08±156.71	16.88±4.05
VICGG-12	719.87±45.83	77.55±6.14	1605.03±140.00	13.19±4.20
VICGG-5	692.43±24.88	75.36±6.35	2125.23±130.59	50.07±2.66
VICGG-29	654.86±24.03	71.69±6.70	1984.74±133.12	32.53±3.39
VICGG-10	649.99±8.80	56.56±8.15	2008.83±132.68	33.26±3.36
VICGG-7	593.63±20.15	50.39±8.74	2163.76±129.90	28.22±3.57
VICGG-37	555.86±5.86	48.99±8.87	2781.90±118.93	50.83±2.63
VICGG-35	524.35±10.01	42.80±9.47	1934.97±134.02	53.27±2.53
VICGG-34	516.36±9.90	38.20±9.91	1824.99±136.01	33.50±3.35
VICGG-32	429.78±18.45	33.36±10.38	1080.81±149.61	26.67±3.64
VICGG-28	428.03±7.56	31.59±10.55	1255.02±146.40	16.39±4.07
VICGG-16	350.09±17.96	ND	1979.93±133.20	44.41±2.90
VICGG-14	318.89±19.84	ND	1576.93±140.51	43.08±2.95
VICGG-38	287.83±14.51	ND	1714.21±138.01	54.42±2.48
B104	ND	ND	ND	ND

ND denotes not detected. Data represent means±SD (n=3 biological replicates)

Table S3 Quantitative RT-PCR analysis of the vip3Aa, cry1Ab, gr79epsps and gat transcript levels in VICGG transgenic maize lines.

Sample	Relative mRNA level			
	vip3Aa	*cry1Ab*	*gr79epsps*	*gat*
VICGG-15	1.56±0.04	1.73±0.05	3.86±1.53	1.45±0.48
VICGG-20	2.77±0.02	3.82±0.18	3.56±2.51	4.06±2.24
VICGG-33	1.00±0.03	1.00±0.04	2.82±0.12	1.00±0.04
VICGG-40	2.18±0.03	2.67±0.11	1.00±0.12	0.01±0.00
VICGG-13	4.00±0.04	4.28±0.13	4.70±0.75	2.78±0.44
VICGG-12	5.19±0.13	6.10±0.18	3.00±0.45	1.57±0.11
VICGG-5	8.55±0.19	8.75±0.27	4.61±0.55	2.83±0.95
VICGG-29	2.08±0.05	2.18±0.06	4.01±0.18	1.07±0.09
VICGG-10	2.19±0.07	2.56±0.15	6.38±1.52	2.42±0.49
VICGG-7	8.17±0.07	9.15±0.26	1.29±0.08	9.04±0.99
VICGG-37	1.38±0.08	1.69±0.04	0.72±0.20	0.16±0.04
VICGG-35	1.87±0.04	2.44±0.15	6.48±2.48	4.56±1.72
VICGG-34	5.77±0.29	9.22±0.49	7.23±1.35	1.47±0.19
VICGG-32	1.25±0.03	1.29±0.08	3.53±0.16	2.10±0.25
VICGG-28	1.78±0.05	1.81±0.09	5.25±1.13	1.87±0.07
VICGG-16	2.39±0.03	2.08±0.08	1.93±0.46	2.40±0.79
VICGG-14	3.5±0.04	3.68±0.11	8.13±2.89	3.19±0.71
VICGG-38	4.18±0.11	4.33±0.31	2.88±0.45	1.02±0.10
B104	ND	ND	ND	ND

ND denotes not detected. Data represent means±SD (n=3 biological replicates).

Table S4 The seedling survival rates of B104 and VICGG-15 at 1, 2 and 4 weeks after treatment with different doses glyphosate.

Sample	Doses (g a.e.ha^{-1})	Seedling rate (%)		
		1 WAT	2 WAT	4 WAT
B104	water	100.00±0.00	100.00±0.00	100.00±0.00
B104	900	0.00±0.00	0.00±0.00	0.00±0.00
B104	1800	0.00±0.00	0.00±0.00	0.00±0.00
B104	3600	0.00±0.00	0.00±0.00	0.00±0.00
VICGG-15	water	100.00±0.00	100.00±0.00	100.00±0.00
VICGG-15	900	100.00±0.00	100.00±0.00	100.00±0.00
VICGG-15	1800	100.00±0.00	100.00±0.00	100.00±0.00
VICGG-15	3600	100.00±0.00	100.00±0.00	100.00±0.00

g a.e.ha^{-1}: grams of equivalent acid per hectare. WAT: weeks after treatment. Data are means±SD (n=3 biological replicates).

Table S5 Agronomic traits of VICGG-15 plants with different doses glyphosate treatment in field.

Sample	Doses (g a.e.ha^{-1})	Plant height (cm)	Ear height (cm)	Ear length (cm)	Ear diameter (cm)	Row numbers per ear	Kernels per row	Bald length (cm)	100-kernel weight (g)	Yield per plant (g)
B104	water	177.20±5.74a	60.20±5.77a	10.81±0.90a	3.94±0.21a	14.6±0.97a	21.5±2.88a	0±0a	20.07±1.78a	52.00±9.15a
VICGG-15	water	178.77±4.35a	59.93±6.82a	10.76±0.92a	3.97±0.16a	14.0±0.94a	22.1±3.87a	0±0a	21.20±1.45a	53.62±6.50a
VICGG-15	900	177.90±5.67a	61.20±6.85a	10.85±0.98a	3.99±0.22a	14.4±0.84a	20.7±3.83a	0±0a	21.63±2.71a	53.61±10.56a
VICGG-15	1800	176.00±6.09a	61.57±6.38a	11.62±1.17a	4.05±0.14a	14.4±1.26a	20.7±3.13a	0±0a	21.44±2.00a	53.50±9.31a
VICGG-15	3600	176.63±4.77a	59.63±7.83a	11.68±1.25a	3.98±0.20a	14.8±1.40a	22.5±4.09a	0±0a	20.01±1.23a	53.36±10.40a

Data are means±SD (n=10). The same letter in column indicate no significant difference. $P>0.05$, one-way ANOVA.
